# Refractory Period Modulates the Spatiotemporal Evolution of Cortical Spreading Depression: A Computational Study

**DOI:** 10.1371/journal.pone.0084609

**Published:** 2014-01-06

**Authors:** Bing Li, Shangbin Chen, Pengcheng Li, Qingming Luo, Hui Gong

**Affiliations:** 1 Britton Chance Center for Biomedical Photonics, Huazhong University of Science and Technology-Wuhan National Laboratory for Optoelectronics, Wuhan, Hubei, China; 2 MoE Key Laboratory for Biomedical Photonics, Department of Biomedical Engineering, Huazhong University of Science and Technology, Wuhan, Hubei, China; University of Southern California, United States of America

## Abstract

Cortical spreading depression (CSD) is a pathophysiological phenomenon, which underlies some neurological disorders, such as migraine and stroke, but its mechanisms are still not completely understood. One of the striking facts is that the spatiotemporal evolution of CSD wave is varying. Observations in experiments reveal that a CSD wave may propagate through the entire cortex, or just bypass some areas of the cortex. In this paper, we have applied a 2D reaction-diffusion equation with recovery term to study the spatiotemporal evolution of CSD. By modulating the recovery rate from CSD in the modeled cortex, CSD waves with different spatiotemporal evolutions, either bypassing some areas or propagating slowly in these areas, were present. Moreover, spiral CSD waves could also be induced in case of the transiently altered recovery rate, i.e. block release from the absolute refractory period. These results suggest that the refractory period contributes to the different propagation patterns of CSD, which may help to interpret the mechanisms of CSD propagation.

## Introduction

Cortical spreading depression (CSD) is a complex pathophysiological phenomenon, which was firstly observed in the cerebral cortex of rabbits in 1944 [1]. It is characterized as an expanding wave that propagates across the cerebral cortex, accompanying with the suppression of neuronal activity, disturbance of ion homeostasis, a negative direct current potential shift and the depression of electrocorticogram [1]–[4]. In the past three decades, the clinical relevance of CSD attracted lots of attention, and CSD was considered to be involved in migraine with aura, epilepsy and ischemic stroke [5]–[7]. A recent study has demonstrated that CSD could trigger headache by activating some neuronal channels [8]. In stroke, the spread of CSD waves could cause secondary damage by deteriorating the damaged tissue and a significant correlation has been discovered between the number of CSD waves and the evolving infarct volume [9], [10]. The spread of CSD wave has been used to monitor progresses of stroke [11], [12]. However, the mechanisms on the propagation of CSD are still not completely understood [3], [13].

One of the striking facts is that the spatiotemporal evolution of CSD wave is varying. Some observations in experiments showed that the individual of successive CSD waves may propagate through the entire observed cortex or bypass parts of the cortex [14]–[17]. The different regional manifestations of CSD waves are thought to be due to the different cytoarchitecture and glia/neuron ratio in experiments [2], [3], [18], [19]. Mathematical models were designed to study the influence of the cortex geometry on the propagation of CSD wave [20], [21]. These models could well interpret the reasons why CSD waves should be blocked in some areas but propagate in other areas in the cortex. However, they could not give an answer to the phenomenon that CSD waves propagate in some areas but then the following CSD waves bypass the same areas. To obtain a better understanding of the propagation patterns of CSD, we have introduced a computational model in this work to investigate the spatiotemporal evolution of CSD, and try to explain the striking phenomenon by the refractory period, which is closely related with the recovery process from CSD. Our results suggest that the refractory period could modulate the different propagation patterns of CSD.

## Methods

**Figure 1:**
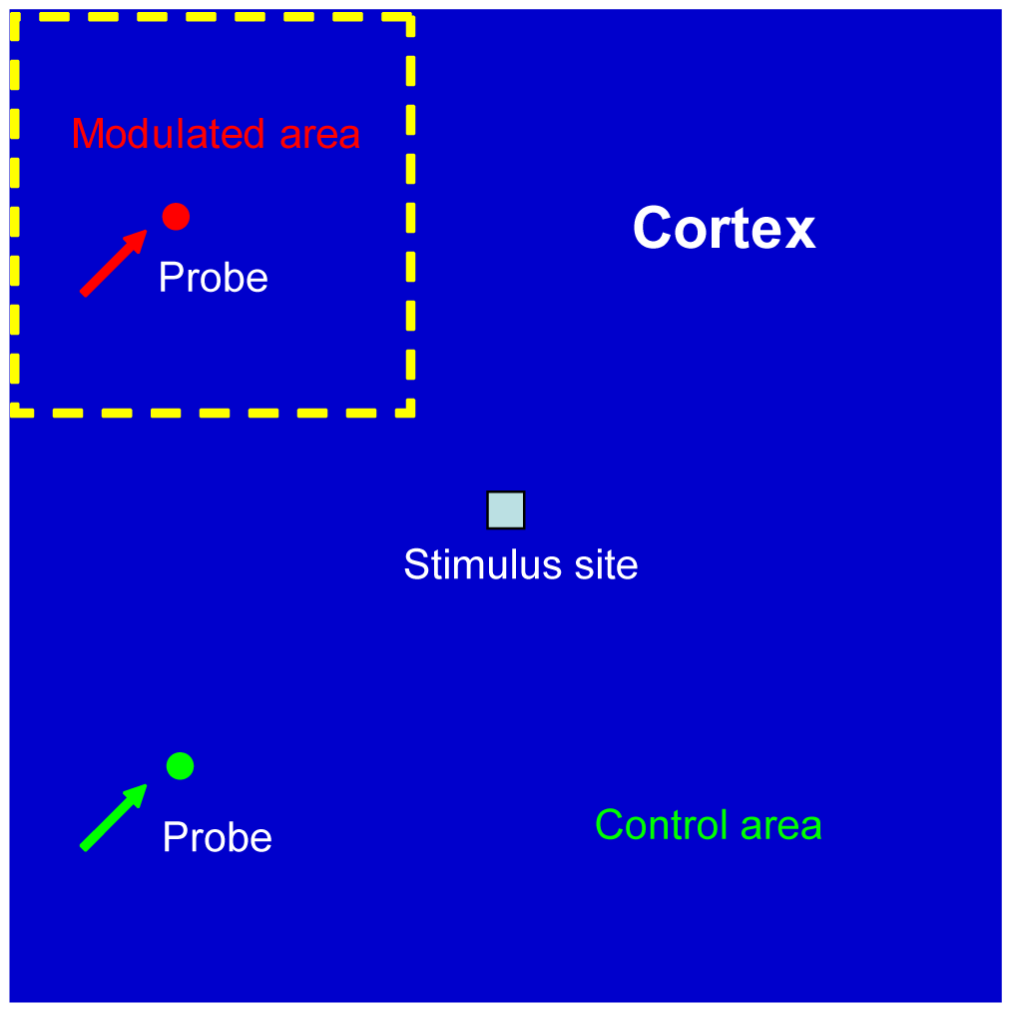
Schematic diagram of the model. The stimulated cortex was divided into two parts, the control area and the modulated area. The modulated area was outlined by a yellow dotted box. Simulated probes were placed in each of the two parts to trace and visualize the spatiotemporal evolution of CSD. CSD waves were evoked in the center of the cortex.



In this model, the cortex was modeled as a 2D sheet (Fig. 1) and CSD waves were induced by elevating the concentrations of extracellular potassium as described previously [10], [22]. As the cortical tissue is invaded by CSD waves, ionic concentrations will be significantly changed. The dynamics of extracellular potassium level is thought to be the main indicator of CSD process [22]–[24]. Here, the extracellular potassium level was characterized by the classic reaction-diffusion equation [22] as following:
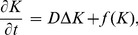
(1)where, *K* is the extracellular potassium level, *D* is the diffusion coefficient of extracellular potassium, and △ is the Laplacian operator in the 2D field. The first term in the right represents as the diffusion of extracellular potassium in a radial fashion, and the second term represents as the reaction process, such as exchange of ions between the neurons and extracellular space. The form of *f(K)* was slightly modified from the reference [22] as below:
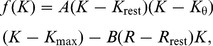
(2)where A, B, *K*_rest_, *K*_θ_, *K*_max_ and *R*_rest_ are constants in this model. *K*_rest_ is the concentration of extracellular potassium in the resting state, *K*_θ_ is the concentration threshold of extracellular potassium beyond which CSD will be evoked, and *K*_max_ is the maximal extracellular potassium level during CSD. Different from the studies which focused merely on the firing of ionic perturbation to explore the spatiotemporal evolution of CSD [20], [21], we also considered the recovery of ions from CSD in this model. *R* is a variable representing as the recovery mechanism and is considered to be closely related with the refractory period in this work.

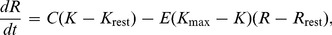
(3)where *C* and *E* are constants. *R*_rest_ is the value of *R* in the resting level. Since the purpose of this study is to explain the varying propagation patterns of CSD wave, all variables in this model are dimensionless, and they could be considered as their quantitative data in relative scales. The definitions and values of the parameters used in this model are shown in Table 1. Except for some parameters referred from the previous reports [10], the others are optimized and fixed using the way described in [25].

**Table 1:** List of the parameters used in this study.

Parameter	Definition	Value	Source
*A*	Coefficient of K^+^ dynamics	−0.54	Fitting
*B*	Coefficient of K^+^ recovery	0.12	Fitting
*C*	Rate for recovery	0.018	Fitting
*D*	Diffusion constant	0.005	Ref. 10
*E*	Rate for recovery	0.03	Fitting
*F*	Factor modulating recovery	0–1	Testing*
*K_rest_*	Resting K^+^	0.03	Ref. 10
*K_θ_*	Threshold K^+^ for CSD	0.2	Ref. 10
*K_max_*	Maximum K^+^ for CSD	1	Ref. 10
*R_rest_*	Resting recovery strength	0.5	Fitting

“Testing*” means the *F* value may be changed in the range of (0,1] among different simulations.

To investigate the influence of the refractory period on the spatiotemporal evolution of CSD, one area in the simulated 2D cortex was chosen as the modulated area where the rate of recovery from CSD was modulated, and the other area, named the control area, had the normal recovery rate. In the modulated area, the recovery rate was slowed by multiplying a factor *F* ranging from 0 to 1 while d*R/dt*<0. By this way, the fastest recovery rate was kept equal to the one in the control area. The regional recovery rate equation was changed into the form as below:
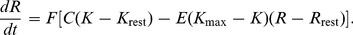
(4)


Two simulated probes were placed in the modulated area and in the control area respectively to trace and visualize the differences in the propagation patterns of CSD in these two areas. The distance from each of the two probes to the stimulus site was equal. In this study, an 80×80 element cortical area was modeled, and the canonical explicit difference method was used to solve the differential equations [26], with a zero-flux boundary condition.

## Results

### CSD Waves Bypassed the Modulated Area

**Figure 2:**
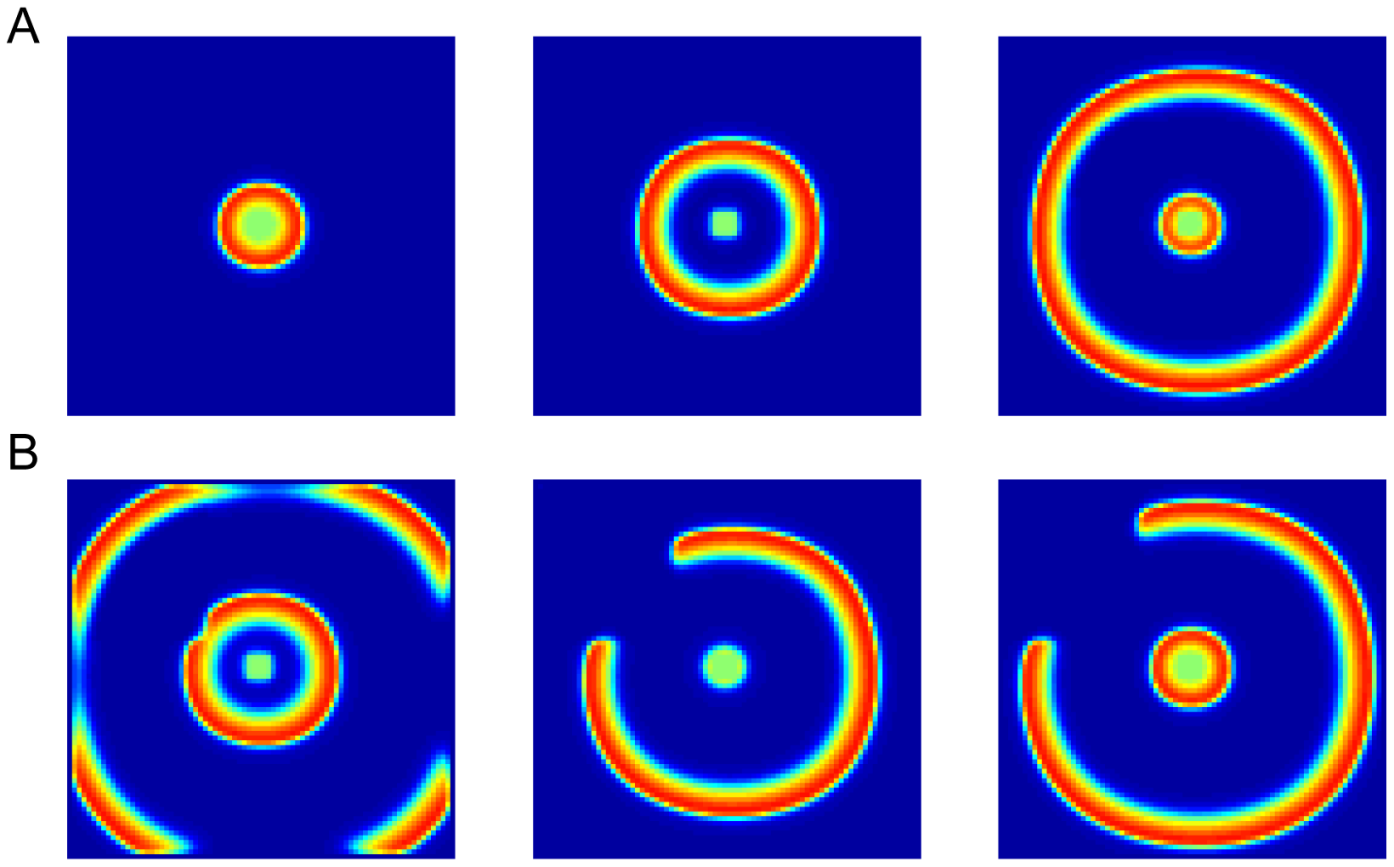
Slow recovery rate blocked the spread of CSD waves into the modulated area. The first CSD wave propagated through the entire cortex (A), but the following one bypassed the modulated area (B). A relevant video was attached as Video S1.

**Figure 3:**
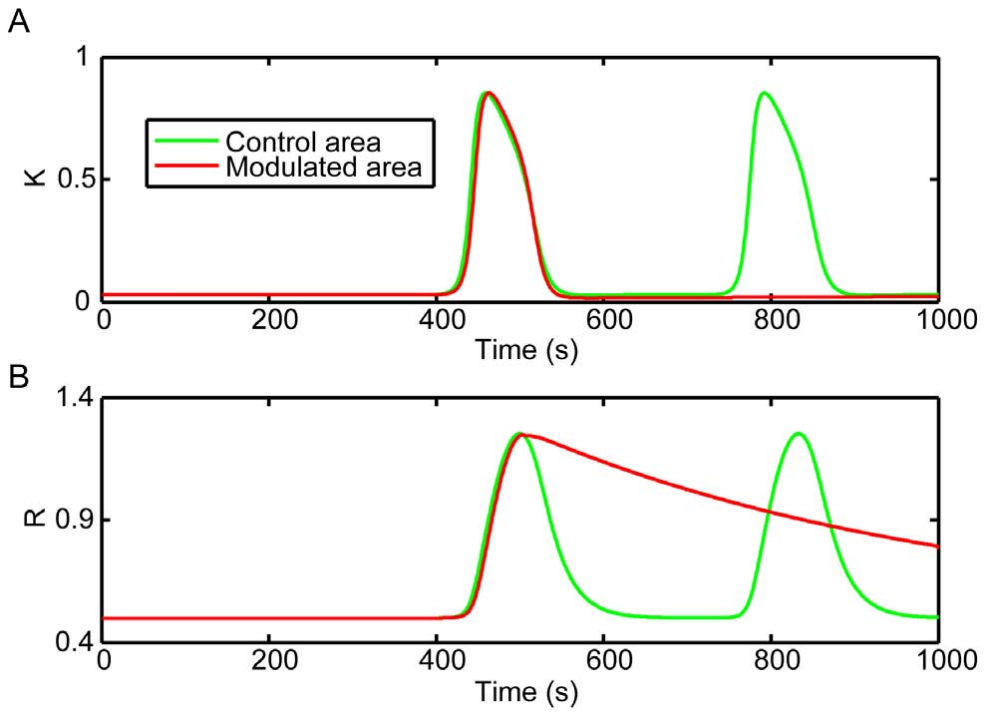
Extracellular potassium level and the recovery variable traced by the simulated probes. (A) Dynamics of extracellular potassium level. (B) Dynamics of the recovery process. The second CSD wave could not spread into the modulated area where the recovery variable was still high.

By simulating the infusion of potassium ions, CSD waves were generated from the stimulus site and propagated towards the surrounding cortex (Fig. 2). As shown in Fig. 2A, the first CSD wave propagated across the entire modeled cortex and showed homogeneous patterns. The probed temporal dynamics of extracellular potassium level in the modulated area was quite similar as that in the control area (Fig. 3A). However, the second CSD wave bypassed the modulated area but propagated into the control area (Figs. 2B and 3A). These results were consistent with the successive, time-varying CSD waves observed in experiments [14]–[17]. Furthermore, compared with that in the control area, the recovery variable *R* remained in a higher status in the modulated area after the second CSD was induced (Fig. 3B). The longer recovery time of *R* coming back to the baseline indicated a longer refractory period (i.e. slow recovery rate of CSD). With exhaustive testing, the bypassing CSD wave happened when the value of the factor *F* is in the range of 0.01 to 0.09. It suggested that the higher *R* value, representing the slow recovery rate, stopped the spread of CSD into the modulated area and affected the propagation range.

### CSD Waves Propagated Slowly in the Modulated Area

**Figure 4:**
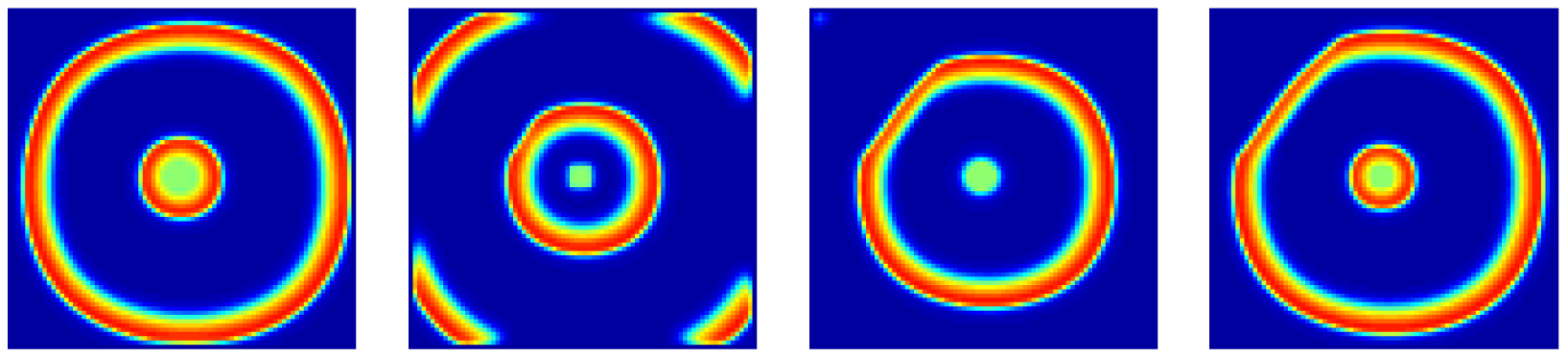
Slow recovery rate reduced the propagation speed of CSD waves in the modulated area. The CSD wave propagated slower in the modulated area where the tissue was incomplete recovery from the previous CSD. A relevant video was attached as Video S2.

**Figure 5:**
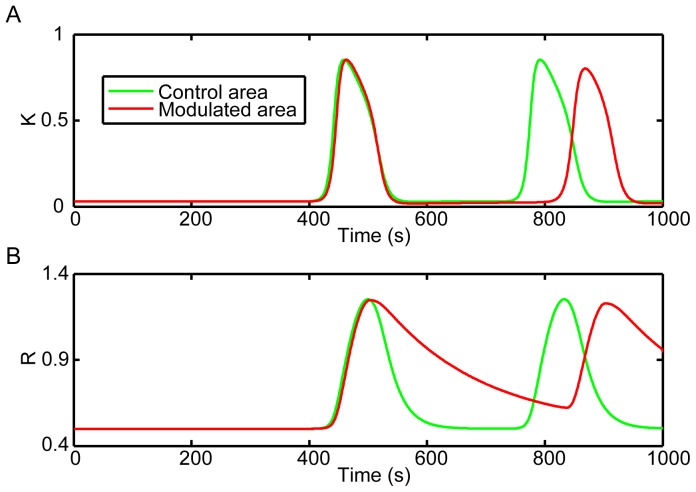
Extracellular potassium level and the recovery variable traced by the simulated probes. (A) Dynamics of extracellular potassium level. (B) Dynamics of the recovery process. CSD propagated slowly in the modulated area where the recovery process was incomplete.

The speed of CSD wave could also be affected by regulating the recovery rate. As shown in Fig. 4 and Fig. 5A, the first CSD wave propagated across the whole cortex at the same speed, while the following CSD wave propagated slower in the modulated area than in the control area. It was easy to see the noncircular pattern of CSD wave and delayed peak time of potassium dynamics in the modulated area. This should be due to the partial recovery of the recovery variable *R* in the modulated area (Fig. 5B), which slowed down the propagation of CSD. Here, the value of the factor *F* was tested in the range of 0.1 to 0.4, and we found that a smaller *F* resulted into a smaller propagation speed in the modulated area. Actually, the phenomenon that CSD propagated with a smaller speed in the incomplete recovery tissue has also been observed in experiments [27], [28]. All of these indicated that the recovery rate could influence the propagation speed of CSD [29].

**Figure 6:**
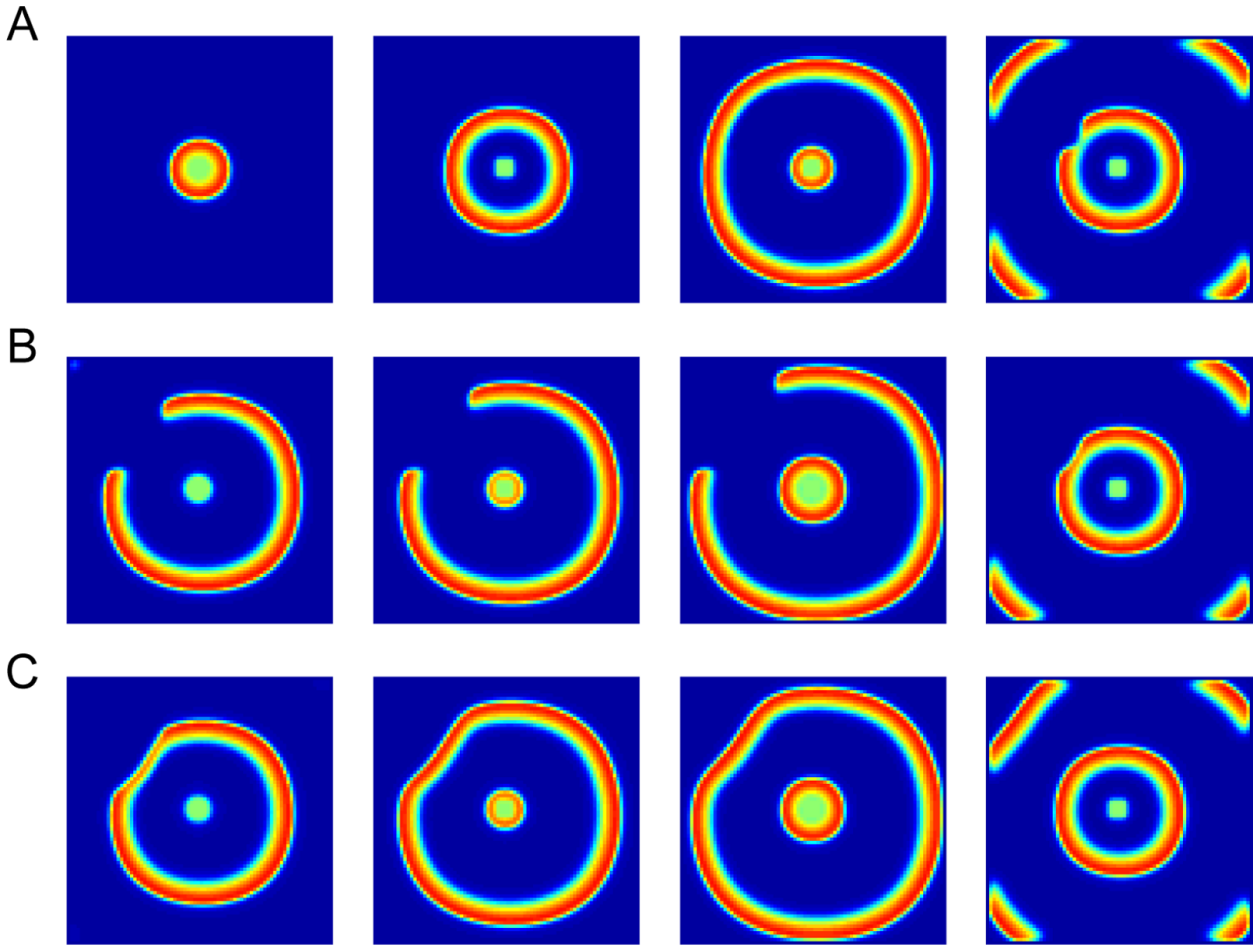
Successive CSD waves with different propagation patterns. (A) The first CSD wave propagated across the whole cortex with homogeneous pattern and speed. (B) The second CSD wave bypassed the modulated area. (C) The third CSD wave propagated into the modulated area with a smaller speed than into the control area. A relevant video was attached as Video S3.

From the results in Fig. 3B and Fig. 5B, they suggested that the spatiotemporal evolution of CSD waves was determined by different recovery rates. Relatively slow recovery rate in the modulated area would reduce the propagation speed of CSD and the much slower recovery rate would block the spread of CSD. It should be noted that as CSD bypassed the modulated area, the recovery process in this area continued and it was possible for the next CSD wave to propagate into the modulated area (Fig. 6).

### Spiral CSD Waves

**Figure 7:**
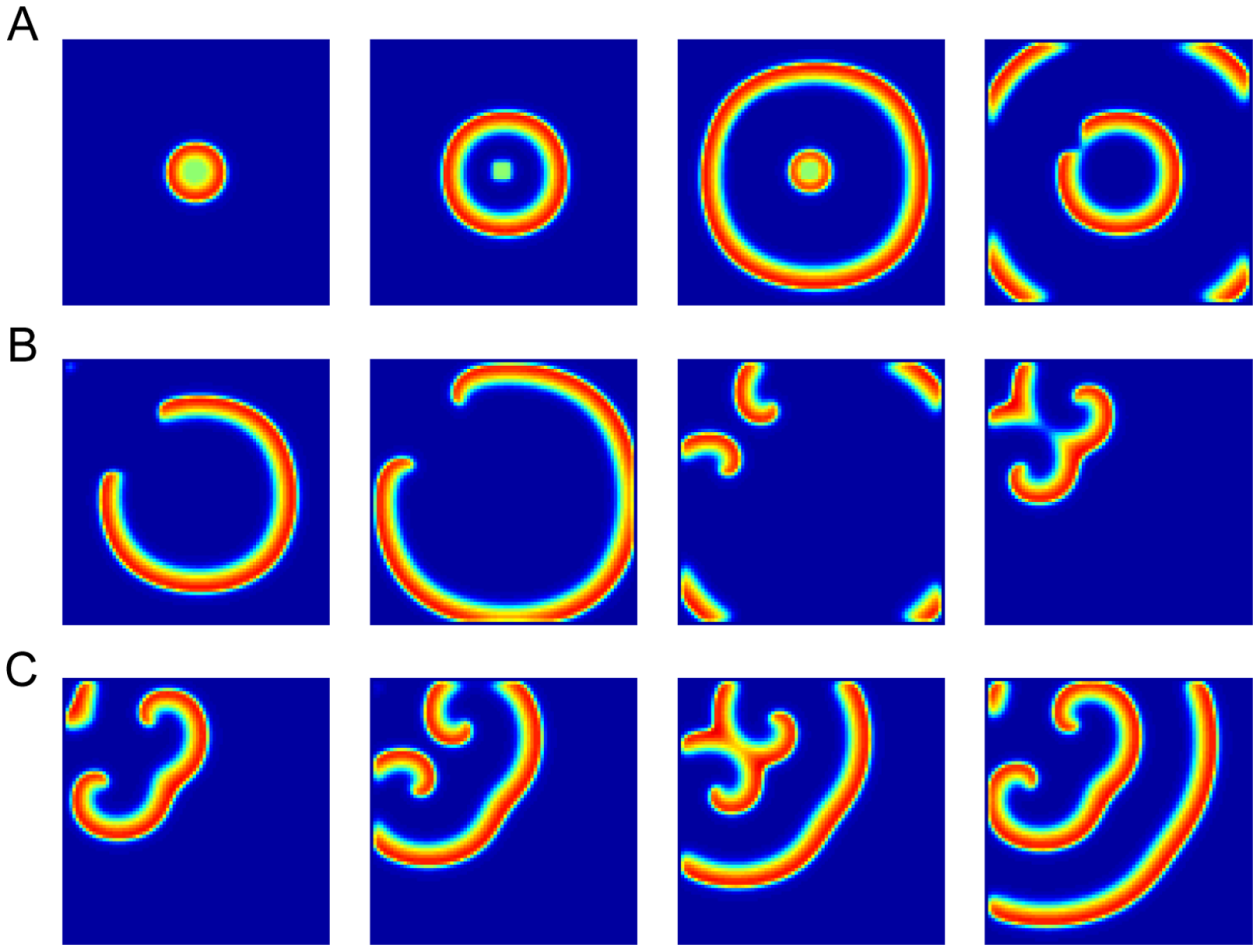
Spiral CSD waves. (A) The first CSD wave propagated across the whole cortex in a radial fashion. (B-C)The laterally opened ends of the successive CSD waves penetrate into the modulated area, circle and generate new CSD waves. A relevant video was attached Video S4.

The spiral CSD waves could also be modeled by modulating the recovery rate in a temporary anodal block style [30]. As shown in Fig. 7B and C, a long segment of the CSD wave was blocked as it reached the modulated area, but the laterally opened ends of the CSD wavefront continued to propagate, turned around and penetrated into the previously blocked modulated area. The two free ends penetrating into the modulated area circled and generated new CSD waves. The two waves collided and formed a larger propagating wavefront. Then the free ends reentered the ever blocked area and expanded repeatedly. These results were quite similar with the spiral and reverberating waves observed in the experimental studies [31]–[33].

## Discussion

CSD has been discovered for almost 70 years [1], but its mechanisms are not fully understood [3], [13]. In this paper, we introduced a simple mathematical model to investigate the spatiotemporal evolution of CSD waves from the perspective of the recovery rate which is considered to be closely related with the refractory period in this work. To mimic the spatiotemporal pattern of CSD waves characterized in experments, this model was specified with two space dimensions simulation [30] rather than the one space dimension studies [34]–[36]. And, this study focused on a series of CSD waves [37], [38], not just on a solitary one [34], [36], [39], [40]. Further more, the varying spatiotemporal evolutions of CSD waves cannot be explained by the influence of the cortex geometry [20], [21] but by the refractory period in this work.

**Figure 8:**
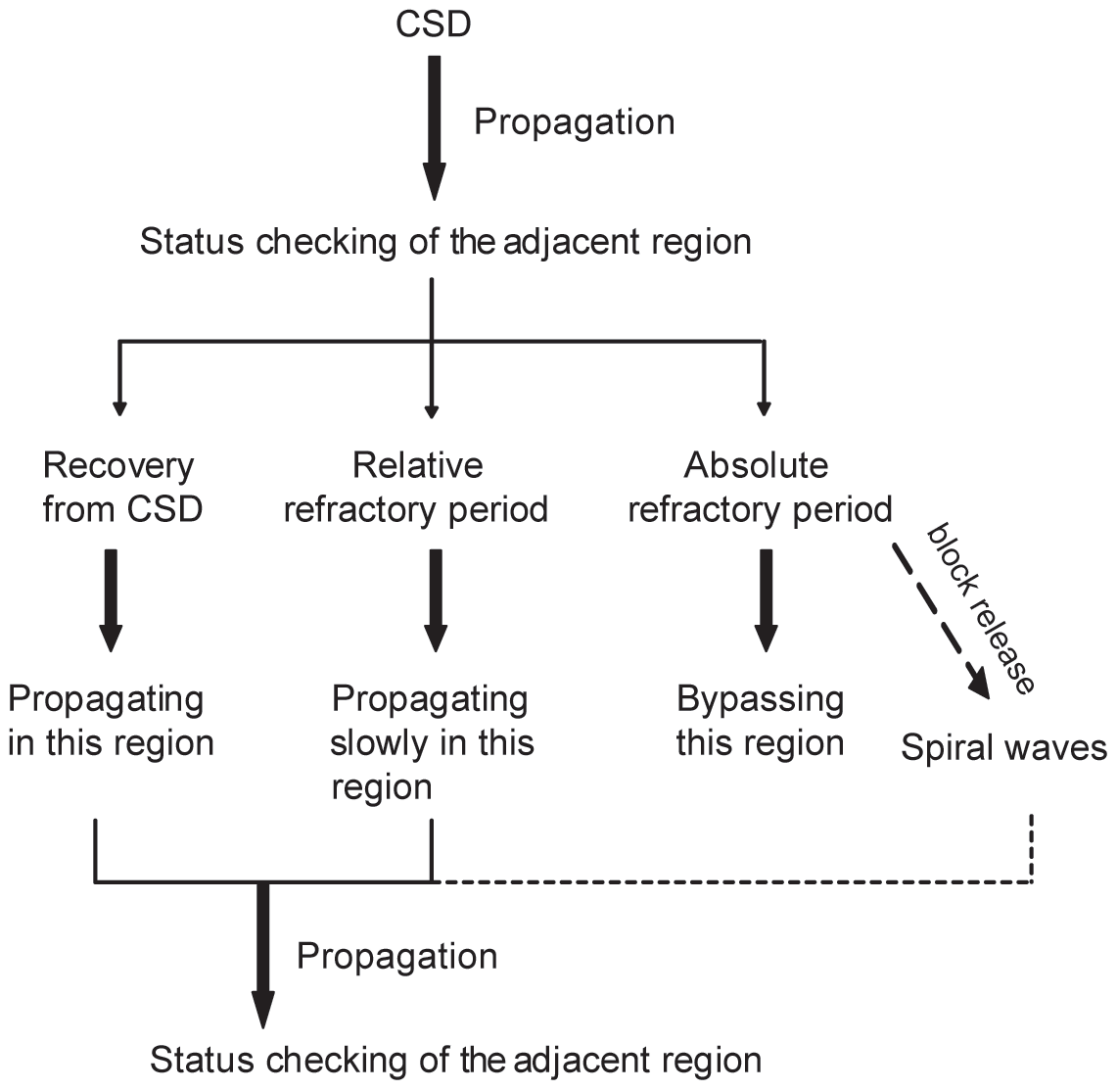
Shematic drawing of the refractoriness effects on CSD wave patterns. CSD wave does not spread in the tissue experiencing the absolute refractory period, or spread with a slower velocity in the tissue experiencing the relative refractory period. But the block release from absolute refractory period may lead to spiral waves.

By reducing the recovery rate from CSD, i.e. increasing the refractory period, our model reproduced the experimental phenomenon that in the successive CSD waves, some waves spread across the entire cerebral cortex, while others bypass parts of the cortex [14]–[17]. Moreover, the speed of CSD wave could also be affected by the decreased recovery rate (Fig. 4). These results suggest that the recovery rate, or the refractory period, has a significant effect on the spatiotemporal evolution of CSD waves. As we known, refractory period includes the absolute and relative refractory period. In this computational study, CSD wave does not spread in the tissue experiencing the absolute refractory period, or spread with a slower velocity in the tissue experiencing the relative refractory period, which is consistent with the experimental results [28], [41], [42]. Interestingly, the block release from absolute refractory period leads to spiral waves. A schematic drawing to summarize the model was shown in Fig. 8.

The different spatiotemporal evolutions of CSD waves indicate the functional inhomogeneity in the cortex [43], [44]. The metabolic status in the cortex could affect the propagation of CSD [10]. By taking the turbulence of ions back to the resting level, Na/K-ATPase plays an important role in the recovery process of CSD, which depends on energy consumption [45]. However, as the energy substances, for example, ATP, are not enough to maintain the activation of Na/K-ATPase, the recovery of ions would be slowed down and then the refractory period is increased [41]. This is not conducive to the propagation of the following CSD waves. Moreover, the energy supply in the cortex is often associated with the blood flow irrigation and oxygen consumption [46], but the distribution of vessels is not uniform in the cerebral cortex. Such a distribution may influence the blood flow irrigation, cause different metabolic rates and contribute to the different propagation patterns of CSD waves in the cortical areas [28].

By modulating the recovery rate from CSD, the spiral and reverberating waves are generated (Fig. 7). Spiral waves have already been reported in the CSD experiments [30]–[33], also in calcium waves in *Xenopus* oocytes [47] and in voltage sensitive dye-characterized spontaneous activities [7]. The cause of these spiral waves is not completely clear. Our results support the notion that the spiral wave is not generated by spiral circuits [7], and further, may help to understand its generation by the refractory period.

Due to the complex nature of CSD, this model only addressed on potassium ion diffusion and recovery. The results suggested that the recovery rate from CSD waves, i.e. the refractory period, modulates the spatiotemporal evolution of CSD waves. A definitive model to cover some identified mechanisms, including ion diffusion, membrane currents, osmotic effects, spatial buffering, gap junctions, metabolic pumps and neurotransmitters et al, has yet to be built [48].

## Supporting Information

Video S1 **A video to show two successive CSD waves.** The second CSD wave bypassed the modulated area.
(AVI)

Video S2 **A video to show two successive CSD waves.** The second CSD wave propagated slowly in the modulated area.
(AVI)

Video S3 **A video to show a series of successive CSD waves.** The second CSD wave bypassed the modulated area and the third CSD wave could propagate into the modulated area but with a smaller speed than in the control area.
(AVI)

Video S4 **A video to show spiral CSD waves.**
(AVI)
