# Correction: Refractory Period Modulates the Spatiotemporal Evolution of Cortical Spreading Depression: A Computational Study

**DOI:** 10.1371/annotation/9b262a84-5035-472d-8386-822970442105

**Published:** 2014-01-15

**Authors:** Bing Li, Shangbin Chen, Pengcheng Li, Qingming Luo, Hui Gong

In the Funding statement, a grant number from the National Natural Science Foundation of China is incorrectly omitted. The Funding Statement should read: 

"This work was supported by the National Natural Science Foundation of China (Grant Nos. 61371014, 81201067), the National High-Tech Research and Development Program of China (863 Program: 2012AA020401), and the Fundamental Research Funds for the Central Universities, HUST: 2013TS038. The funders had no role in study design, data collection and analysis, decision to publish, or preparation of the manuscript."

